# Metronidazole-induced encephalopathy in a patient with cirrhosis

**DOI:** 10.1016/j.radcr.2023.10.001

**Published:** 2023-10-28

**Authors:** Xinyu Ji, Ke Xuan Li, Leonardo Furtado Freitas, Philippe Huot

**Affiliations:** a Faculty of Medicine, McGill University, Montreal, QC, Canada; b Department of Diagnostic Radiology, Clinical Neuroradiology Fellow, McGill University, Montreal, QC, Canada; c Neurodegenerative Disease Group, Montreal Neurological Institute-Hospital (The Neuro), Montreal, QC, Canada; d Department of Neurology & Neurosurgery, McGill University, Montreal, QC, Canada; e Movement Disorder Clinic, Division of Neurology, Department of Neuroscience, McGill University Health Centre, Montreal, QC, Canada

**Keywords:** Antibiotic, Encephalopathy, Metronidazole, MRI, Neurotoxicity

## Abstract

Metronidazole is a commonly used antibiotic with anaerobic bacterial, protozoal, and microaerophilic bacterial coverage. Encephalopathy and peripheral neurotoxicity are rare but known adverse events with prolonged metronidazole use, which can be difficult to distinguish from other causes of delirium in acutely ill patients. Definitive diagnosis can be made by brain magnetic resonance imaging (MRI), which often reveals symmetric bilateral hypersignal demyelination lesions typically involving the dentate nuclei, splenium of the corpus collosum, midbrain, dorsal medulla, and pons. This case report describes a 72-year-old male presenting with altered mental status and neurological deficits after prolonged metronidazole use for bacteremia with spondylodiscitis, with full clinical and neuroradiological resolution upon appropriate diagnosis and drug cessation. Neuroradiologists play an indispensable role in recognizing this rare and poorly understood manifestation.

## Introduction

Metronidazole-induced encephalopathy is a rare but documented adverse effect of this commonly used antibiotic [1]. The clinical presentation is characterized by cerebellar symptoms, peripheral neuropathy, seizures, and altered mental status [1,2]. The differential diagnosis of neurocognitive changes in an acutely ill patient is often broad, and metronidazole toxicity should be considered, particularly for patients on prolonged treatment courses. Neuroimaging with magnetic resonance imaging (MRI) provides definitive diagnosis.

We report the observation of a 72-year-old patient with new dysphagia, dysarthria, and confusion following 8 weeks of metronidazole treatment for complicated spondylodiscitis. Importantly, we highlight the hallmark neuroradiological findings of metronidazole toxicity and demonstrate full resolution on repeat imaging after drug cessation.

## Case report

A 72-year-old male known for atrial fibrillation, ischemic stroke, alcoholic cirrhosis, and chronic obstructive pulmonary disease was recently found to have L5-S1 spondylodiscitis and *Bacteroides fragilis* bacteremia. He underwent washout, L4-S1 fusion, and was treated with ertapenem, which was stepped down to oral metronidazole and moxifloxacin. Eight weeks following his discharge, he returned to the emergency department with progressive altered mental status, decreased appetite, and generalized weakness. His initial evaluation was notable for dysarthria, dysphagia, and both visual and auditory hallucinations.

**Fig. 1:**
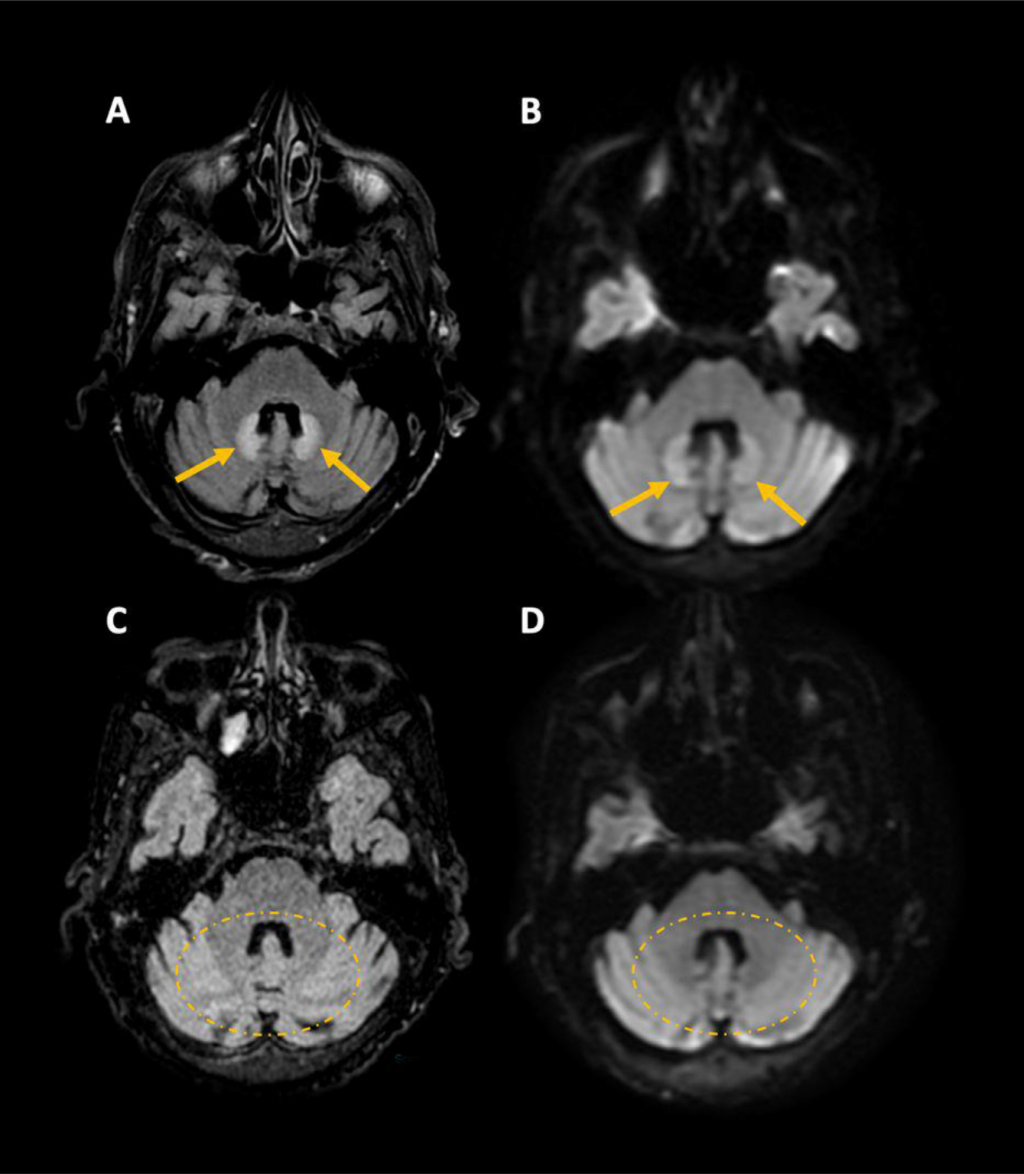
Axial FLAIR (A and C) and DWI (B and D) sequences of a brain MRI. At the initial presentation, there was symmetric FLAIR/diffusion hyper signal in the dentate nuclei bilaterally (arrows). Follow-up MRI 3 months after discontinuation of the medication demonstrated complete resolution of the lesions, without sequelae (dashed circles).

A delirium workup, including complete blood count, metabolic panel, and biochemistry, was unremarkable. To rule out stroke, a computed tomography (CT) of the head without contrast and CT-angiogram were performed and showed no acute ischemia or hemorrhage. However, the MRI of the brain demonstrated symmetric hypersignal T2/FLAIR/diffusion without restriction on apparent diffusion coefficient map of both dentate nuclei and hypersignal T1 of the globi pallidi, subthalamic nuclei and substantia nigra, bilaterally (Fig. 1A and B). In this clinical context, the findings were suspicious for metronidazole-induced encephalopathy.

Following these radiological findings, metronidazole was discontinued and changed to piperacillin-tazobactam. During the following week, the patient showed gradual improvement of his cognitive function. With discontinuation of the metronidazole, his repeat MRI performed 2.5 months later (Fig. 1C and D) showed complete resolution of the diffusion-weighted imaging and FLAIR signal abnormalities in the dentate nuclei, supporting our initial diagnosis of metronidazole induced encephalopathy.

## Discussion

Metronidazole is a commonly used antibiotic with anaerobic bacterial, protozoal, and microaerophilic bacterial coverage. It is known to easily cross the blood-brain barrier and is metabolized by the liver. Its excretion is primarily through bile, and about 6 to 18% of unmetabolized metronidazole is excreted by the liver [1,3,4]. Despite a relatively safe pharmacokinetic profile, with common adverse effects including nausea, it may also cause QT prolongation and disulfiram-like reactions with alcohol. More rarely, metronidazole induced encephalopathy can occur in an estimated 0.2% of patients [2,5].

The mechanism by which metronidazole causes encephalopathy remains not fully elucidated. Hypothesized pathways involve interference with ribonucleic acid protein synthesis leading to axonal degeneration, Purkinje cell damage [6] and free radical formation [7]. Common clinical manifestations include cerebellar dysfunction in the form of dysarthria, ataxia, altered mental status, peripheral neuropathy, and seizures [4]. Older patients appear to be more susceptible to seizures, while younger patients more commonly present with acute mental status [8]. The average time of onset for neurological adverse effects has been reported to be 30 days, although this varies based on the literature [5,8].

Metronidazole-induced radiological findings can occur in the cerebellar dentate nuclei, colossal splenia, and dorsal pons [5,8,9], although imaging findings without cerebellar involvement have also been reported. Salari et al. (2022) shared a case of metronidazole-induced encephalopathy associated with diffuse white matter and corpus callosum lesions with diffusion restriction [10]. Few other medications, such as vigabatrin, are documented to have similar localization of toxicity on neuroimaging [9]. This patient's concomitant history of alcoholic cirrhosis likely contributed to decreased metronidazole elimination. Case reports suggest these lesions often fully resolve upon metronidazole cessation, while some cases describe irreversible neurological sequelae [2,6,8,11].

Alternative differential diagnoses of bilateral dentate nuclei hyperintensity include, but are not limited to, Krabbe's disease, cerebro-tendinous xanthomatosis, Wilson's disease, Fahr's syndrome, L 2-hydroxyglutaric aciduria, progressive multifocal leukoencephalopathy, and lead poisoning [9] – none of which were consistent with the patient's clinical history. Notably, the prolonged course of metronidazole may have also contributed to the adverse effect [1].

Currently, there is no clinical consensus on the use of any additional pharmaceutical agents to improve the clinical recovery of patients who suffer from metronidazole-induced encephalopathy. Prior case reports suggest potential benefit of steroid therapy and diazepam for symptom resolution in human and animal cases [12,13]. In 2019, Li et al. reported successfully administering high-dose methylprednisolone pulse therapy (500 mg/d) – concomitantly given with thiamine and vitamin B12 – to prevent rapid neurological deterioration. However, no similar reports are available on the benefit of steroid therapy. Further studies are needed to make formal treatment recommendations. Currently, consensus management includes metronidazole discontinuation and supportive care. In reversible cases, documented clinical recovery period spans three to sixteen weeks [5,8].

## Conclusion

In summary, we discussed a rare case of metronidazole-induced encephalopathy, reviewed its clinical and radiological manifestations, and demonstrated an example of clinical and radiological improvement after the cessation of the drug. Metronidazole toxicity should be considered in patients presenting with acute neurological deficits after a prolonged course of treatment, especially in patients with underlying diseases placing them at risk of reduced drug metabolism. Diagnostic imaging should be performed with neurology and neuroradiology guidance, and management success should be verified with repeat neuroimaging 1-2 months following drug cessation.

## Patient consent

Written informed consent for the writing and publication of this case report was obtained from the patient presented.
